# Low Postseroconversion CD4^+^ T-cell Level Is Associated with Faster Disease Progression and Higher Viral Evolutionary Rate in HIV-2 Infection

**DOI:** 10.1128/mBio.01245-18

**Published:** 2019-01-08

**Authors:** Angelica A. Palm, Philippe Lemey, Marianne Jansson, Fredrik Månsson, Anders Kvist, Zsófia Szojka, Antonio Biague, Zacarias José da Silva, Sarah L. Rowland-Jones, Hans Norrgren, Joakim Esbjörnsson, Patrik Medstrand

**Affiliations:** a Department of Laboratory Medicine, Lund University, Lund, Sweden; b Department of Microbiology and Immunology, Rega Institute, KU Leuven—University of Leuven, Leuven, Belgium; c Department of Translational Medicine, Lund University, Lund, Sweden; d Department of Clinical Sciences Lund, Lund University, Lund, Sweden; e Department of Biochemistry and Molecular Biology, University of Debrecen, Debrecen, Hungary; f National Public Health Laboratory, Bissau, Guinea-Bissau; g Nuffield Department of Medicine, NDM Research Building, University of Oxford, Oxford, United Kingdom; Medical School, University of Athens

**Keywords:** disease progression, human immunodeficiency virus, viral evolution

## Abstract

A positive correlation between virus evolutionary rate and disease progression has been shown for human immunodeficiency virus type 1 (HIV-1) infection. Much less is known about HIV-2, the second causative agent of AIDS. We analyzed 528 HIV-2 *env* V1-C3 sequences generated from longitudinal plasma samples that were collected from 16 study participants during a median observation time of 7.9 years (interquartile range [IQR], 5.2 to 14.0 years). Study participants were classified as faster or slower disease progressors based on longitudinal CD4^+^ T-cell data. The HIV-2 evolutionary rate was significantly associated with CD4^+^ T-cell levels and was almost twice as high among the faster progressors as among the slower progressors. Higher evolutionary rates were accounted for by both synonymous and nonsynonymous nucleotide substitutions. Moreover, slow disease progression was associated with stronger positive selection on HIV-2/SIVsm (simian immunodeficiency virus infecting sooty mangabey) surface-exposed conserved residues. This study demonstrated a number of previously unknown characteristics linking HIV-2 disease progression with virus evolution. Some of these findings distinguish HIV-2 from HIV-1 and may contribute to the understanding of differences in pathogenesis.

## Abstract (precis)

The relationship between HIV evolution and disease progression is fundamental to our understanding of HIV immune control and vaccine design. There are no clear definitions for faster and slower HIV-2 disease progression and for the relationship of the rate of progression with HIV-2 evolution. To address the hypothesis that viral evolution is correlated with disease progression in HIV-2 infection, we determined faster and slower disease progression based on follow-up data from a prospective cohort of police officers in Guinea-Bissau. The analysis showed that although the CD4^+^ T-cell level and the decline in the level were independently associated with progression to AIDS, only the CD4^+^ T-cell level or a combined CD4^+^ T-cell level/decline stratification was associated with the rate of HIV-2 evolution. The HIV-2 evolutionary rate was almost twice as high among the faster progressors as among the slower progressors. Importantly, this report defines previously unknown characteristics linking HIV-2 disease progression with virus evolution.

## INTRODUCTION

It has been estimated that one million to two million people worldwide are infected with HIV-2 (1). Similarly to HIV-1, HIV-2 causes AIDS, but with lower rates of transmission, CD4^+^ T-cell decline, and disease progression (2). Despite similar levels of integrated viral DNA (proviral DNA), the plasma viral RNA burden (viral load) at comparable CD4^+^ T-cell counts is significantly lower in HIV-2 infections than in HIV-1 infections, suggesting either that HIV-2 is associated with a lower rate of replication or that HIV-2 is more susceptible to immune control (2–8). However, despite the lower level of chronic immune activation in HIV-2 infection, both viruses elicit several immune responses that may modulate disease progression, e.g., neutralizing antibodies and cytotoxic T lymphocytes (2).

The natural history of HIV-2 infection was not known in detail until recently, and cohort data often lack information on the estimated time of HIV-2 infection, precluding assessment of the true rates of HIV-2 disease progression and of the dynamics of CD4^+^ T-cell change and plasma viral load during infection (9, 10). In a recent study, we showed that most individuals infected by HIV-2 progress to disease but at a lower rate than for HIV-1 (11). Moreover, the role of CD4^+^ T-cell dynamics in HIV-2 infection was shown to be a strong clinical predictor of disease progression. Thus, both HIV-1 disease progression and HIV-2 disease progression are associated with CD4^+^ T-cell decline and early initial postseroconversion CD4^+^ T-cell levels (11–15).

Both HIV-1 and HIV-2 evolve rapidly due to high mutation rates, high replication rates, and fast generation times (16). This results in extensive genetic variability both within and between infected individuals. The association between viral evolutionary rate (5, 17) and disease progression has been studied extensively for HIV-1, and most studies have suggested that these parameters are positively correlated (18). Much less is known about HIV-2 intrapatient evolution; whereas some researchers have reported that HIV-2 has a lower evolutionary rate than HIV-1, others have reported the opposite (5, 17, 19–21). However, no correlation has been found between the virus evolutionary rate and variations in levels of CD4^+^ T-cells over time in HIV-2 infection (5, 20). Importantly, those studies were performed on proviral DNA or on RNA obtained from virus propagated in culture, which may not reflect the circulating virus populations. To the best of our knowledge, differences in HIV-2 evolutionary rates between groups with different rates of disease progression have not been investigated.

Here, we aimed to determine whether faster disease progression and slower disease progression are differentiated by rates of decline of and levels of CD4^+^ T cells in HIV-2 infection, similarly to what has been previously suggested to be the case in HIV-1 infection (12, 14), and investigated the association between the disease progression rate and the evolutionary dynamics of HIV-2.

## RESULTS

### Study population.

The HIV-2 *env* V1-C3 region was successfully reverse transcribed and amplified from 53 plasma samples that had been collected longitudinally from 16 study participants of a well-described cohort of police officers in Guinea-Bissau (22, 23). Inclusion criteria and clinical characteristics are presented in Materials and Methods (see also Table S1 and S2 at https://dochub.com/patrik-medstrand/zqp8z8/supplemntaryinformation_palm_mbio_ver2_180822?dt=Nx2HKD__sa_SKWC-XzVE). The median observation time from inclusion until the last registered visit for the 16 included individuals was 19.2 years (interquartile range [IQR], 15.0 to 20.8 years). A median of seven clones from each time point were sequenced, and the median time between the collection dates of the first and last amplified patient-specific samples was 7.9 years (IQR, 5.2 to 14.0 years). Among the 528 sequences, 119 putative recombinant sequences were removed, leaving 409 sequences for evolutionary analyses. Phylogenetic subtype analysis showed that all sequences belonged to HIV-2 group A. Moreover, all sequences from each individual formed distinctive monophyletic clades in the phylogeny, indicating that sequences from the study participants were not subjected to superinfection, coinfection, mix-up during sample handling, or contamination during the laboratory procedures (see Fig. S1 at https://dochub.com/patrik-medstrand/zqp8z8/supplemntaryinformation_palm_mbio_ver2_180822?dt=Nx2HKD__sa_SKWC-XzVE).

### Classification of individuals as faster and slower progressors based on longitudinal CD4^+^ T-cell dynamics.

**FIG 1:**
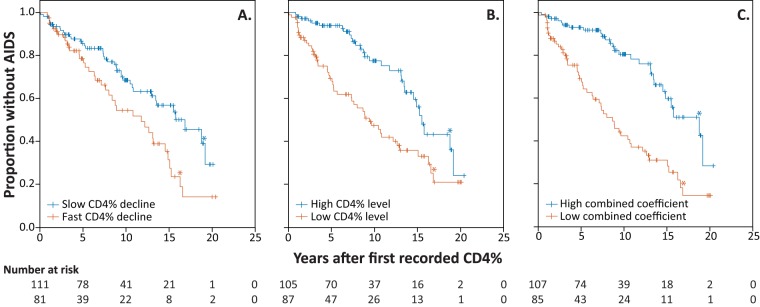
Kaplan-Meier curves of AIDS-free time. Three stratifications were explored (fast and slow progressors were defined by having values above or below the mean values from all participants for each stratification). (A) CD4% decline rate. (B) CD4% level at the midpoint in time between the first and last recorded CD4% values. (C) Combined effect of CD4% decline rate and CD4% level. Tick marks indicate participants with censored data. Asterisks indicate the time points in each group when five participants were still at mortality risk and at risk of developing AIDS. The numbers of individuals at risk are given below the figure panels at 5-year intervals. Data from faster progressors are shown in vermillion, and data from slower progressors are shown in blue.

To assess the link between HIV-2 evolutionary estimates and disease progression, we classified the study participants into groups of faster disease progressors and slower disease progressors based on longitudinal CD4^+^ T-cell dynamics from the entire HIV-2-infected population of the Guinea-Bissau police cohort. To determine if faster disease progression can be differentiated from slower disease progression by CD4^+^ T-cell decline and level in cases of HIV-2 infection also, as had been previously suggested for HIV-1 infection (12, 14), we analyzed the time to the appearance of AIDS from the first recorded percentages of CD4 (CD4%) for all HIV-2-infected individuals in the cohort with two or more CD4% measurements (*n* = 192). Three stratifications were used, and faster and slower progressors were defined as those patients whose CD4% values were above and below the mean of the values determined for all participants for each stratification, respectively (Fig. 1). The first stratification, referred to as the CD4% decline rate, was based on individual coefficients of regression for CD4%. Eighty-one participants were classified as faster progressors (mean CD4% decline, 2.7% per year [standard deviation {SD}, 2.6]) and 111 as slower progressors (mean CD4% increase, 0.5% per year [SD, 1.6]). The median time to AIDS from the first recorded CD4% value was 11.7 years (95% confidence interval [CI], 7.3 to 16.1 years) for faster progressors with a faster CD4% decline and 16.8 years (CI, 12.3 to 21.3 years) for slower progressors with a slower CD4% decline (*P* = 0.008 [log rank test]) (Fig. 1A). The second stratification, referred to here as the CD4% level, was determined as the CD4% level at the midpoint in time between the first and last recorded CD4% levels using the regression line generated in the first stratification. Eighty-seven participants were classified as faster progressors (mean CD4% level, 21.0% [SD, 4.3%]) and 105 as slower progressors (mean CD4% level, 35.0% [SD, 5.7%]). The median time to AIDS from the first recorded CD4% value was 9.4 years (95% CI, 6.7 to 12.1 years) for faster progressors with a low CD4% level and 15.5 years (95% CI, 14.3 to 16.6 years) in slower progressors with a high CD4% level (*P* < 0.001 [log rank test]) (Fig. 1B). In the third stratification, referred to as the combined coefficient, the CD4% decline rate and CD4% level were combined to have equal influences on the stratification (see Materials and Methods). Eighty-five participants were classified as faster progressors (combined coefficient below the mean) and 107 as slower progressors (combined coefficient above the mean). The median time to AIDS from the first recorded CD4% value was 8.6 years (95% CI, 6.5 to 10.8 years) for faster progressors with a low combined coefficient and 18.7 years (95% CI, 13.6 to 23.8 years) for slower progressors with a high combined coefficient (*P* < 0.001 [log rank test]) (Fig. 1C). Interestingly, only 50% of the participants were consistently classified as faster or slower progressors in the stratifications (see Table S3 at https://dochub.com/patrik-medstrand/zqp8z8/supplemntaryinformation_palm_mbio_ver2_180822?dt=Nx2HKD__sa_SKWC-XzVE). A Cox proportional-hazards model with CD4% decline and level as discrete covariates (according to the stratifications described above) showed that CD4% decline and level were independently associated with disease progression rate (*P* = 0.007 for CD4% decline and *P* < 0.001 for CD4% level [Wald test]; the independence of these covariates was determined by a nonsignificant interaction term between the covariates corresponding to *P* = 0.85 [Wald test]).

### The HIV-2 evolutionary rate is associated with CD4% level and combined coefficient but not with CD4% decline alone.

Since all three stratifications described above were significantly linked to the rate of disease progression, we decided to assess differences in virus evolutionary parameters for all three stratifications (Table 1). Model evaluation within the hierarchical phylogenetic modeling (HPM) framework indicated that a strict clock with a constant size demographic model generally fitted our data best for both the nucleotide and codon models (see Materials and Methods and Text S1 at https://dochub.com/patrik-medstrand/zqp8z8/supplemntaryinformation_palm_mbio_ver2_180822?dt=Nx2HKD__sa_SKWC-XzVE for details). The overall mean evolutionary rate of the V1-C3 region was 23.5 × 10^−3^ codon substitutions/site/year (95% highest posterior density [HPD] interval, 20.3 × 10^−3^ to 26.6 × 10^−3^) (Table 2). No association was found between evolutionary rate and disease progression when individuals were classified as faster or slower progressors based on CD4% decline rate (Bayes factor [BF] = 0.3) (Table 2). In contrast, when individuals were classified based on either CD4% level or the combined coefficient, the mean evolutionary rate was significantly higher in faster progressors (28.6 × 10^−3^ codon substitutions/site/year; 95% HPD, 24.2 × 10^−3^ to 33.5 × 10^−3^) than in slower progressors (14.9 × 10^−3^ codon substitutions/site/year; 95% HPD, 12.2 × 10^−3^ to 17.6 × 10^−3^) (BF = 20.3) (Table 2). Similar differences were found for the nucleotide substitution rate (BF = 0.3 for association with CD4% decline rate and BF = 17.5 for association with CD4% level or the combined coefficient) (see Table S4 at https://dochub.com/patrik-medstrand/zqp8z8/supplemntaryinformation_palm_mbio_ver2_180822?dt=Nx2HKD__sa_SKWC-XzVE).

**TABLE 1:** Disease progression classifications of all individuals, based on three different stratifications for CD4% (*n* = 16)

Individual	CD4% decline rate/year		CD4% level		Combined coefficient	
	Value^*a*^	Progressor^*b*^	Value^*a*^	Progressor^*b*^	Value^*a*^^,^^*c*^	Progressor^*b*^
DL3405	−1.46	Slow	33.95	Slow	1.00	Slow
DL3542	0.69	Fast	32.73	Slow	0.74	Slow
DL2051	1.61	Fast	31.11	Slow	0.62	Slow
DL2876	0.55	Fast	28.91	Slow	0.67	Slow
DL3654	−0.15	Slow	27.74	Slow	0.70	Slow
DL2533	−1.10	Slow	23.96	Slow	0.68	Slow
DL2316	1.10	Fast	23.45	Fast	0.50	Fast
DL2794	−0.11	Slow	20.99	Fast	0.53	Fast
DL3941	3.40	Fast	20.65	Fast	0.29	Fast
DL2381	1.17	Fast	20.62	Fast	0.44	Fast
DL2335	0.58	Fast	20.41	Fast	0.47	Fast
DL3647	0.73	Fast	19.63	Fast	0.44	Fast
DL3646	0.33	Slow	19.32	Fast	0.46	Fast
DL3222	−0.88	Slow	19.25	Fast	0.53	Fast
DL3740	−0.69	Slow	17.57	Fast	0.48	Fast
DL2386	1.61	Fast	16.29	Fast	0.32	Fast

a Data represent individual values for the three different stratifications.

b Individuals with values above or below the mean values determined for all individuals were grouped as faster or slower progressors, respectively.

c The combined coefficient values represent a combination of CD4% decline rate and CD4% level. The combined coefficient values were obtained by transforming the CD4% decline rate values and CD4% level values, setting them to comparable scales, and subsequently multiplying them.

**TABLE 2:** Evolutionary rates (10^−3^ codon substitutions/site/year) in the V1-C3 *env* regions determined using a strict clock hierarchical phylogenetic model^*a*^

Genetic region	All individuals^*c*^	CD4% stratification					
		CD4% decline rate			CD4% level and the combined coefficient^*b*^		
		Fast progressors^*c*^	Slow progressors^*c*^	Bayes factor^*d*^	Fast progressors^*c*^	Slow progressors^*c*^	Bayes factor^*d*^
V1-C3	23.5 (20.3-26.6)	24.7 (20.1-29.6)	21.9 (19.1-24.9)	0.3	28.6 (24.2-33.5)	14.9 (12.2-17.6)	20.3
V1V2	29.5 (25.1-34.2)^*a*^	30.1 (24.6-36.0)	28.8 (23.7-34.0)	0.3	35.4 (28.9-42.2)	19.6 (14.8-24.7)	11.8
C2	18.0 (15.2-20.7)^*a*^	18.6 (15.3-21.9)	16.7 (13.9-19.5)	0.3	21.5 (17.6-25.7)	12.0 (9.2-15.2)	28.4
V3	21.2 (17.0-25.7)^*a*^	21.6 (16.5-26.8)	20.8 (15.7-25.9)	0.3	24.1 (18.0-30.6)	16.5 (11.0-22.2)	2.4
C3	26.6 (22.6-31.1)^*a*^	27.0 (22.0-32.4)	26.0 (21.6-30.6)	0.2	30.4 (24.3-27.0)	20.2 (15.2-25.8)	6.1

a *P* values for Wilcoxon signed rank tests for comparisons of rates between regions were as follows: for V1V2 versus C2, <0.001; for V1V2 versus V3, 0.002; for V1V2 versus C3, 1; for C2 versus V3, 0.005; for C2 versus C3, <0.001; for V3 versus C3, <0.001.

b The combined coefficient values represent a combination of CD4% decline rate and CD4% level.

c Data correspond to 10^−3^ codon substitutions per site per year (95% highest posterior density interval).

d Bayes factor (BF) support for association between codon substitution rate and disease progression (faster versus slower progressors). BF >3 was considered evidence of a significant association.

### Higher evolutionary rate in the HIV-2 V1V2 and C3 region than in the C2 and V3 region.

To determine if the evolutionary rates were similar in different regions of *env*, we partitioned the data set into four well-defined regions: V1V2, C2, V3, and C3. The evolutionary rate was higher in both the V1V2 and C3 regions than in the C2 and V3 regions, respectively (Table 2) (*P < *0.05 [Wilcoxon signed rank test with Bonferroni correction for all pairwise comparisons]). No significant difference was found between faster and slower progressors in any of the four V1-C3 regions when individuals were stratified by CD4% decline rate (BF values ranged from 0.2 to 0.3). In contrast, moderate to strong associations between evolutionary rate and disease progression were found in the V1V2 (BF = 11.8), C2 (BF = 28.4), and C3 (BF = 6.1) regions for individuals grouped according to CD4% level or the combined coefficient. A weak association was also noted in the V3 region (BF = 2.4).

Since the CD4% level and the combined coefficient stratifications resulted in identical groupings (Table 1) and all analyses indicated a strong association between evolutionary rate and CD4% level—in contrast to the absence of associations between evolutionary rate and CD4% decline rate—only results based on the CD4% level stratification are presented for the subsequent analyses.

### V1-C3 of HIV-2 *env* evolves under conditions of negative selection.

Next, we hypothesized that the differences in evolutionary rate between the progressor groups could have originated in parameters influencing selection pressure or viral replication. We therefore estimated the ratio of nonsynonymous substitution rates to synonymous substitution rates (the dN/dS rate ratio), which indicates whether a gene or site had been subjected to positive selection (dN/dS > 1) or to negative selection (dN/dS < 1) or had evolved neutrally (dN/dS = 1). We found global negative selection over the entire V1-C3 region (dN/dS rate ratio = 0.56; 95% HPD interval, 0.49 to 0.63), with no statistical differences apparent between faster and slower progressors (BF = 1.3) (see Table S5 at https://dochub.com/patrik-medstrand/zqp8z8/supplemntaryinformation_palm_mbio_ver2_180822?dt=Nx2HKD__sa_SKWC-XzVE). The region-specific analyses showed that the V1V2 and C3 regions evolved neutrally (dN/dS rate ratios, 1.27 [95% HPD interval, 0.84 to 1.80] and 0.94 [95% HPD interval, 0.70 to 1.22], respectively) whereas the C2 and V3 regions had been subjected to strong negative selection (dN/dS rate ratios, 0.22 [95% HPD interval, 0.18 to 0.27] and 0.41 [95% HPD interval, 0.27 to 0.57], respectively). No significant differences were found in dN/dS rate ratios between the progressor groups in the V1V2, V3, or C3 regions (BF values of <3 for all regions), whereas a moderately higher level of negative selection was found in the C2 region among faster progressors than among the slower progressors (BF = 3.7) (see Table S5 at https://dochub.com/patrik-medstrand/zqp8z8/supplemntaryinformation_palm_mbio_ver2_180822?dt=Nx2HKD__sa_SKWC-XzVE).

### Higher nonsynonymous and synonymous substitution rates in faster progressors than in slower progressors.

**FIG 2:**
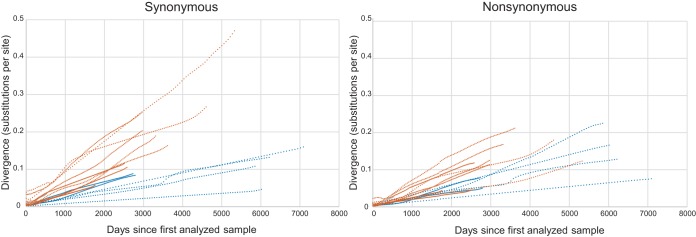
Synonymous and nonsynonymous divergence over time. Data represent accumulated synonymous and nonsynonymous divergences over time in the V1-C3 *env* region of HIV-2 for each study participant. The divergence from the level seen with the first analyzed sample from each study participant is shown. Data from faster progressors are shown in vermillion, and data from slower progressors are shown in blue.

Whereas the dN/dS rate ratio provides an estimate of the overall selective pressure, analyses of expected nonsynonymous (E[N]) and expected synonymous (E[S]) substitution rates can add detailed information concerning replication and selection pressure (17). The E[N] and E[S] divergences were estimated and plotted as accumulated divergence rates over time from the first analyzed sample (Fig. 2; see also Table S6 at https://dochub.com/patrik-medstrand/zqp8z8/supplemntaryinformation_palm_mbio_ver2_180822?dt=Nx2HKD__sa_SKWC-XzVE). The HIV-2 divergence rates increased in a relatively linear manner during the asymptomatic phase of infection, and viral populations in faster progressors accumulated both nonsynonymous and synonymous substitutions faster than those in slower progressors. The median E[N] rate ranged from 3.0 × 10^−3^ to 6.6 × 10^−3^ nucleotide [nt] substitutions/site/year for faster progressors and from 1.4 × 10^−3^ to 4.6 × 10^−3^ nt substitutions/site/year for slower progressors (*P = *0.005 [two-tailed Mann-Whitney U test {M-W}]). The median E[S] rate ranged from 4.5 × 10^−3^ to 11.2 × 10^−3^ nt substitutions/site/year for faster progressors and from 1.1 × 10^−3^ to 4.0 × 10^−3^ nt substitutions/site/year for slower progressors (*P < *0.001 [M-W]). These results are in line with the higher overall viral evolutionary rates for faster progressors than for slower progressors seen in the HPM analyses and clarify why no statistically significant differences were found in dN/dS rate ratios.

### Higher number of conserved sites under conditions of positive selection in slower HIV-2 disease progression.

**FIG 3:**
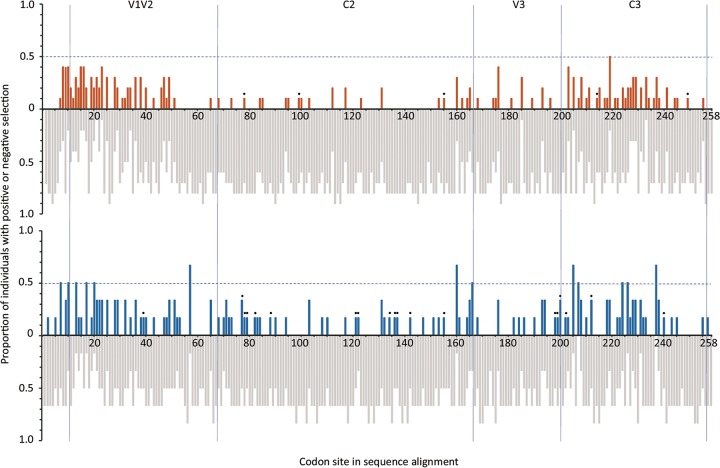
Codon-specific selective pressure. The selective pressures at each codon site of the sequence alignment of the *env* V1-C3 region of HIV-2 for faster and slower disease progressors are indicated. The proportions (*y* axis) with positive selection (vermillion or blue) or negative selection (gray) at codon sites of the sequence alignment (*x* axis) are shown for faster progressors (upper graph) and slower progressors (lower graph). Filled black circles indicate codons conserved between HIV-2/SIVsm strains associated with positive selection. Vertical lines divide the fragment into *env* regions V1V2, C2, V3, and C3.

Although the analysis described above indicated the presence of general negative selection across the entire V1-C3 region, we hypothesized that a number of specific residues could have been subject to positive selection. To assess this hypothesis, we used a Renaissance counting procedure to estimate the dN/dS rate ratio at each codon site (Fig. 3). Only a low proportion of sites showed signatures of positive selection, and the proportions of positively selected sites were not significantly different between the viral populations of faster and slower progressors across the V1-C3 region (7% and 11%, respectively; *P = *0.503 [M-W]) or in the V1-C3 regions in separate analyses (see Table S7 at https://dochub.com/patrik-medstrand/zqp8z8/supplemntaryinformation_palm_mbio_ver2_180822?dt=Nx2HKD__sa_SKWC-XzVE). While the numbers of sites under positive selection were similar in the faster and slower progressors in the V1V2, V3, and C3 regions, slower progressors had more sites under positive selection in the C2 region than faster progressors (35 versus 20 sites) (*P = *0.026 [two-tailed Fisher’s exact test {FET}]) (Fig. 3). Since it has been shown that the HIV-2 C2 and C3 regions are exposed similarly to the corresponding HIV-1 regions and are under negative selection, we hypothesized that the effects of positive selection on amino acids subjected to structural and functional constraints would negatively impact viral fitness (24). We therefore defined the amino acids critical to viral fitness as those amino acids that were conserved between HIV-2 and the simian immunodeficiency virus (SIV) from which HIV-2 originated through cross-species transmission (i.e., SIVsm, the SIV infecting sooty mangabeys [Cercocebus atys]) and compared the ratios of positive selection of viral lineages between slower and faster progressors (25). Among the 246 amino acids in the V1-C3 regions, 84 were conserved between SIVsm and HIV-2 (see Table S8 at https://dochub.com/patrik-medstrand/zqp8z8/supplemntaryinformation_palm_mbio_ver2_180822?dt=Nx2HKD__sa_SKWC-XzVE). Comparisons of the numbers of conserved HIV-2/SIVsm sites under positive selection showed that slower progressors had more conserved sites under positive selection than faster progressors (20 versus 5 sites (*P = *0.002 [FET]) in the V1-C3 region. The differences between the slower and faster progressors with respect to positive selection at conserved sites were most highly pronounced in the C2 region (12 versus 3 sites, respectively) (*P = *0.021 [FET]), while such differences were not observed in the V1V2, V3, and C3 regions. To confirm previous observations that C2 is well exposed on the HIV-2 *env* gene, we used the published structural data of HIV-2 gp125 to visualize the amino acids in the V1-C3 region (24, 26). This analysis indicated that the majority (15 of 22) amino acids associated with positive selection mapped to exposed surfaces on HIV-2 gp125 (see Fig. S2 at https://dochub.com/patrik-medstrand/zqp8z8/supplemntaryinformation_palm_mbio_ver2_180822?dt=Nx2HKD__sa_SKWC-XzVE). Moreover, the surface accessibility of amino acids within the V1-C3 regions showed that the positively selected sites were associated with residues with solvent-exposed surfaces in the C2 region (29 of 68 amino acids) (*P = *0.040 [FET]).

## DISCUSSION

The relationship between HIV evolution and disease progression is fundamental to our understanding of HIV immune control and vaccine design. We recently showed an association between CD4^+^ T-cell decline/CD4^+^ T-cell level and HIV-2 disease progression rate (11). Here we defined relatively faster and slower HIV-2 disease progression using these associations and dissected the associations between HIV-2 evolutionary dynamics and disease progression. Studies addressing these associations for HIV-2 infection have been limited (5, 17, 20, 27). In HIV-2 infection, disease in many patients progresses slowly, but in some the advance is as fast as that in HIV-1 infection (28–30). The reasons for this marked heterogeneity are currently not known, but mechanisms similar to those of HIV-1 infection may be involved (11, 31). To address the hypothesis that viral evolution is associated with disease progression in HIV-2 infection also, we first determined stratifications for relatively faster and slower disease progression based on follow-up data from the entire prospective cohort of police officers in Guinea-Bissau. The analysis showed that although CD4^+^ T-cell level and decline were independently associated with progression to AIDS, the effect size was largest for the CD4% level or the combined CD4% level/decline stratifications. This observation is in line with previous reports showing that the CD4^+^ T-cell level at corresponding time points after infection may be a better marker for both HIV-1 and HIV-2 disease progression rates than CD4^+^ T-cell decline (11, 12, 14). Moreover, only the CD4^+^ T-cell level or the combined CD4^+^ T-cell level/decline stratifications were associated with the evolutionary rate of HIV-2. This observation suggests that the postseroconversion CD4^+^ T-cell level is associated with the rate of disease progression (32), whereas the rate of CD4^+^ T-cell decline during chronic infection can be viewed as an additive component influencing progression in combination with the initial CD4^+^ T-cell levels. The reasons for and mechanisms that determine the variability of CD4^+^ T-cell decline range from genetic and biological factors to physiological factors (33, 34). In those with a low postseroconversion level of CD4 cells, progression to AIDS is faster (and the time to AIDS shorter) than among those with a higher postseroconversion level of CD4 cells (11). It is possible that events that occur during acute infection dictate the initial postseroconversion levels of CD4 T-positive (T^+^) cells in HIV-2 infection also (11, 32). Thus, a broader assessment of disease progression may provide additional understanding of the mechanisms that drive the disease pathogenesis.

Many HIV-2-infected individuals remain nonprogressors with low viral loads during the course of infection, and HIV-2 sequences can be obtained only from individuals with detectable plasma viral loads (35). Hence, HIV-2-infected individuals without detectable viremia cannot be assessed in studies of HIV-2 evolution in plasma. However, our assay had a detection limit of 12 RNA copies/ml plasma, indicating that even slow progressors with low viral loads (<50 copies/ml plasma) could be detected.

A strong association between HIV-2 evolutionary rate and disease progression was found in all the studied genetic subregions, except for V3. For HIV-1, the flanking V3 region is known to be highly exposed and immunodominant (36). By contrast, the V3 region of HIV-2 has been suggested to be more highly concealed from the immune system and to be the least entropic and positively selected part of the C2-C3 region (24). Our findings of lower evolutionary rates and stronger purifying selection in the C2 and V3 regions support previous suggestions that the immune response of HIV-2-infected individuals may be more highly directed to other regions of the HIV-2 envelope (27, 37, 38).

Our HIV-2 evolutionary rate estimates are in the range of what has been reported by others, although direct comparisons are difficult due to differences in the *env* regions analyzed and to the use of different phylogenetic models (17, 20, 21). In a previous study of HIV-1 subtype B based on a similar methodological approach, the evolutionary rate of the HIV-1 V1-V3 region was estimated to be approximately twice as high as our estimates for HIV-2 (39). The uncertainty of how the HIV-2 evolutionary rate compares with the rate of HIV-1 highlights the need for a direct comparison of HIV-1 evolution to HIV-2 evolution in the same population using the same approaches. Previous studies of HIV-2 intrahost evolution have been based on limited numbers of individuals and time points. The generally low viral loads among HIV-2-infected individuals continue to present a large technical challenge and are likely to have contributed to the paucity of intrahost HIV-2 evolutionary studies (6–8).

In a stratified analysis, we found that both nonsynonymous and synonymous substitutions accumulated at a higher rate in faster progressors than in slower progressors. This result suggests generally faster replication rates and shorter generation times for virus populations in faster progressors and is in line with previous reports of increased virus replication rates among immunosuppressed individuals infected with HIV-2 (40). It is possible that increased replication rates can reduce the generation time in intrahost virus populations and can lead to higher rates of neutral evolution. This has also been suggested to explain the association between disease progression and synonymous substitution rates in HIV-1 infection (17, 41).

Previous studies have demonstrated that the *env* gene is under purifying selective pressure overall in both HIV-1 and HIV-2 infection, with a few irregularly distributed positively selected sites (42, 43). Comparisons between HIV-1 disease progressor groups have suggested that slow-disease progressors are associated with a higher number of positively selected sites (44). Similarly to HIV-1 data, we identified a few positively selected sites in the majority of HIV-2 slow progressors. However, the mean dN/dS rate ratios and the proportions of positively selected sites did not differ between slower and faster progressors. Instead, we found that slow HIV-2 disease progression was associated with a higher level of positive selection on a selected number of surface-exposed residues conserved between HIV-2/SIVsm. It is tempting to speculate that slow progressors may elicit a stronger immune response to highly surface-exposed conserved residues, which may in turn impact viral fitness, since such conserved amino acids are likely to have a functional and structural impact on envelope functions (24, 27, 45). If true, our findings would be consistent with the concept that hosts who mount a stronger immune response against the infecting virus have greater numbers of positively selected sites and progress to AIDS at a lower rate, which is reflected by higher postseroconversion CD4^+^ T-cell levels (44). In line with this, Bohl et al. showed that mutations of conserved residues of HIV-2 envelope resulted in poor envelope function (46).

In conclusion, our analyses show a strong association between HIV-2 evolutionary rate and disease progression as determined by CD4% levels. Overall negative selection was demonstrated in the analyzed HIV-2 *env* fragment, with the proportion of positively selected sites in the range of what has been shown for HIV-1. Interestingly, slow disease progression among HIV-2-infected individuals was associated with higher levels of positive selection on residues conserved between HIV-2 and SIVsm, which may indicate generally reduced viral fitness among these viral variants. Our findings provide new insights into the associations between pathogenesis and intrahost evolution of HIV-2. Still, more studies on how the dynamics of disease progression rate is shaped by the molecular evolution of HIV-2 are warranted. Further knowledge of HIV-2 pathogenesis and comparisons between HIV-1 and HIV-2 will be important to reveal fundamental differences in how these two viruses cause immunodeficiency.

## MATERIALS AND METHODS

### Study population.

This study included individuals from a large cohort of police officers in Guinea-Bissau, West Africa, which was formed in 1990 (22, 23). At inclusion, and at follow-up visits scheduled with an interval of 12 to 18 months, individuals were examined and a plasma sample was collected. The civil war in 1998 to 1999 temporarily (from June 1998 until the end of 2002) ended inclusion, but annual visits of previously included individuals were resumed in July 2000. The cohort was followed routinely until February 2011, when the cohort was closed. In September 2013, however, selected individuals from the cohort were asked to participate in a special sampling round, including a clinical examination and collection of a plasma sample. In early 2006, the police cohort was included in the national antiretroviral therapy (ART) program which was introduced into Guinea-Bissau in 2005. HIV testing was performed at the National Public Health Laboratory (LNSP), Bissau, as previously described (22). The CD4^+^ T-cell count and CD4% were determined at each follow-up visit after infection (14, 22, 23). In the absence of commercial HIV-2 RNA assays, HIV-2-infected patients are generally monitored by CD4^+^ T-cell levels, particularly in areas of endemicity. Consequently, viral load measurements have not been included as a standard procedure in Guinea-Bissau, preventing us from a comprehensive analysis of viral load data in this study.

The cohort includes 438 seroincident and seroprevalent HIV-2-infected individuals, 83 of whom had an estimated date of seroconversion, defined as the midpoint between the last HIV-2 seronegative sample collection date and the first seropositive sample collection date. Individuals with long follow-up series, including both CD4% measurements and available plasma samples, were considered for inclusion in the study. Our initial goal was to conduct this study by including only individuals with estimated dates of infection. Thus, amplification of viral RNA was attempted on plasma samples from seroincident individuals from whom three or more longitudinal plasma samples were available (*n* = 19). As expected, due to the generally low viral loads in HIV-2 infections, amplification was successful for only a minority of samples and seven individuals with two or more successfully amplified longitudinal samples could be included in the study. We therefore decided to also include seroprevalent individuals (i.e., individuals who were HIV-2 infected already at enrollment) in the study. Due to the anticipated difficulties in amplifying HIV-2 RNA, only individuals with more than six available plasma samples were considered for inclusion. Amplification was attempted on samples from 19 individuals, and successful amplification of two or more longitudinal samples was achieved for 9 individuals. Taking the data together, amplification was attempted on samples from 38 individuals, where 16 individuals fulfilled the inclusion criterion of the availability of two or more amplified longitudinal samples (total, 53 samples). The majority of the plasma samples were collected from the 16 participants included in this study during the asymptomatic phase of infection, defined by a CD4^+^ T-cell count of >200 cells/μl, CD4% of >14, and a lack of clinical AIDS symptoms (WHO stage 4 and CDC stage C [47, 48]). However, seven samples from six individuals were collected after the individuals had developed AIDS, and two samples from two individuals were collected after the individuals had initiated antiretroviral therapy (see Table S2 at https://dochub.com/patrik-medstrand/zqp8z8/supplemntaryinformation_palm_mbio_ver2_180822?dt=Nx2HKD__sa_SKWC-XzVE). Baseline characteristics of the 16 individuals are presented in Table S1 at https://dochub.com/patrik-medstrand/zqp8z8/supplemntaryinformation_palm_mbio_ver2_180822?dt=Nx2HKD__sa_SKWC-XzVE, and successfully amplified samples are listed in Table S2 at https://dochub.com/patrik-medstrand/zqp8z8/supplemntaryinformation_palm_mbio_ver2_180822?dt=Nx2HKD__sa_SKWC-XzVE.

The 16 included individuals were subsequently stratified as faster or slower progressors using three different parameters: CD4% decline rate, CD4% level, and a combined coefficient. The combined coefficient was achieved by combining the CD4% decline rate and the CD4% level. Since these values were quantified on different scales and since the CD4% decline rate could be either positive or negative, we transformed the values before combining them. The two variables were transformed and rescaled to have equal levels of influence on the combined coefficient. More specifically, to account for negative CD4% decline rates, all rates were transformed to positive rates with the corresponding relative difference. Next, CD4% decline rates and CD4% levels were rescaled to include the same extrema. Individual combined coefficients were then determined for each study participants by multiplying the rescaled CD4% decline rates and CD4% levels. There were no differences in age between the groups at the time of the first HIV-2-positive sample (data not shown).

### Amplification and sequencing.

Viral RNA was extracted from patient plasma samples using an miRNeasy microkit (Qiagen, Stockholm, Sweden) with minor modifications to the manufacturer’s instructions. Briefly, 200 μl of plasma was disrupted in 2,000 μl QIAzol and loaded onto an RNeasy MinElute Spin column in the presence of 15 μg carrier RNA (Qiagen, Stockholm, Sweden). DNA was removed using an on-column DNase treatment (Qiagen, Stockholm, Sweden), and purified RNA was eluted in 22 μl RNase-free H_2_O. An approximately 935-bp fragment that included the complete V1-C3 region of *env* (nt 6986 to 7920 in the BEN reference sequence]; GenBank accession number M30502) was amplified using 9.5 μl eluted RNA in a SuperScript III One-Step reverse transcription-PCR (RT-PCR) system with Platinum *Taq* DNA polymerase followed by a seminested PCR approach using Platinum *Taq* High Fidelity (Invitrogen, Copenhagen, Denmark). Primers KH2_OF (5′-GAGACATCAATAAAACCATGTGTC-3′) and TH2_OR (5′-TTCTGCCACCTCTGCACTAAAGG-3′) were used for One-Step PCR, and primers KH2_OF and KH2_OR (5′-ACCCAATTGAGGAACCAAGTCA-3′) were used for nested PCR (5, 42). Following the initial cDNA synthesis performed for 30 min at 50°C, the PCR conditions were identical for One-Step PCR and the nested PCR: initial denaturation for 2 min at 94°C; 40 cycles of 15 s at 94°C, 30 s at 50°C, and 1 min at 68°C; and a final elongation step for 5 min at 68°C. The sensitivity of the PCR was found to be 12 RNA copies/ml plasma as determined by a dilution series performed with an electron microscopy-counted HIV-2 particle (Advanced Biotechnologies, Eldersburg, MD, USA). Molecular cloning of the amplified fragments using a pCR2.1 TOPO cloning system (Invitrogen) was performed by BaseClear BV (Leiden, The Netherlands), and 12 individual clones were routinely picked for subsequent sequencing on both strands using conventional M13 primers.

Sequences were manually edited using CodonCode Aligner v1.5.2 (CodonCode Corporation, Dedham, MA, USA) and aligned in MEGA5 using the Clustal algorithm (49). Sequences of poor quality and sequences containing stop codons were removed from the analysis. Parts of the sequence that were difficult to align were removed in full codons to preserve an open reading frame, resulting in a final alignment length of 774 bp. The alignment spanned the last 30 bp in the 3′ end of the C1 region, the entire V1-C3 region, and the first 6 bp in the 5′ end of the V4 region. To analyze different subregions, the fragment was partitioned as follows: V1V2, bp positions 31 to 201 (positions 7040 to 7318 in the BEN reference sequence); C2, positions 202 to 498 (7319 to 7615); V3, positions 499 to 600 (7616 to 7717); C3, positions 601 to 768 (7718 to 7891).

### Survival analysis.

Kaplan-Meier analyses were performed for progression time to AIDS. Cases that did not reach AIDS during follow-up were right censored at their last clinical examination date. Statistical comparisons were performed by the log rank test. A Cox proportional-hazards model was applied to determine independency of covariates (as defined by nonsignificant interaction terms between covariates).

### Phylogenetic analysis.

Putative intrapatient recombinant sequences were identified by the pairwise homoplasy index (PHI) test using an exhaustive and iterative search algorithm and were then removed from the data set (50) (the Perl script for the iterative search is available from the authors upon request). Maximum likelihood (ML) phylogenetic trees were reconstructed using the inferred model, GTR +I + G, with Garli v2.0 (51). Statistical support for internal branches was determined by ML-based approximate likelihood ratio test (aLRT) Shimodaira-Hasegawa (SH)-like branch support, as implemented in PhyML 3.0 (52). SH values of >0.9 were considered statistically significant (53). For subtype analysis, our data set was aligned with reference sequences of the major HIV-2 subtypes (downloaded from the Los Alamos Sequence Database [54]) in MEGA5 using the Clustal algorithm (49), followed by phylogenetic analysis.

### Evolutionary rate analysis.

Analyses of HIV-2 evolutionary rates were performed in BEAST v1.7.5 (55) by reconstructing Bayesian rooted and time-measured phylogenetic trees. Unless otherwise stated, all analyses were performed by running a Markov chain Monte Carlo (MCMC) analysis for 50 × 10^6^ generations, with sampling performed every 2,500 to 5,000 generations. Convergence was determined by calculation of effective sample sizes (ESS) of >100 and inspection of traces, as assessed in Tracer v1.6 (available from http://beast.bio.ed.ac.uk/software/tracer/), following removal of 10% after burn-in. In explorative analyses, we evaluated different models to find the one that best fit our data (see Table S9 at https://dochub.com/patrik-medstrand/zqp8z8/supplemntaryinformation_palm_mbio_ver2_180822?dt=Nx2HKD__sa_SKWC-XzVE). For each individual, the nucleotide substitution rates were estimated using two different clock models (strict and uncorrelated lognormal relaxed clocks), two different demographic models (Bayesian skyline plot and constant size), and either partitioned (1st plus 2nd and 3rd) or nonpartitioned codon positions. Nucleotide substitution rates were estimated using the Hasegawa, Kishino, and Yano (HKY) substitution model (56) with gamma-distributed rates. Exploratory analyses were performed by running a single analysis of 50 × 10^6^ MCMC generations as described above. All subsequent analysis were performed in duplicate, and the results were combined in LogCombiner v1.7.5 after the removal of 10% after burn-in (55). Phylogenetic trees were visualized in Figtree v1.40 (available from http://tree.bio.ed.ac.uk/software/figtree/).

Hierarchical phylogenetic modeling (HPM) with fixed effects, as implemented in BEAST v1.7.5, was used to compare differences in evolutionary rate between progressor groups (55, 57). HPM allows simultaneous analysis of sequence data from multiple individuals. Information of evolutionary parameters is pooled across populations or individuals through hierarchical prior specification, resulting in a shrinkage effect of the variation when data are sparse for study participants. In addition, fixed effects across a group of individuals (in this case, the faster and slower progressor groups) can be included to test and quantify differences between them. Analyses were performed using a strict or uncorrelated lognormal relaxed clock model with a constant population size model as the tree prior. Both nucleotide substitution rates (HKY) and codon substitution rates (determined using the GY94 codon model) were estimated (56, 58). In a region-specific analysis, the data set was partitioned into the *env* V1V2, C2, V3 and C3 regions and the evolutionary rate was estimated for all regions simultaneously by HPM (strict clock, constant size, and GY94 codon model). Evolutionary rates were compared between groups as assessed by Bayes factors (BFs). A BF value of >3 was considered to represent a significant association (59).

### Absolute rates and divergence plots.

The ratio of nonsynonymous and synonymous substitution rates (dN/dS rate ratio) has been widely used as an indicator of selection and molecular adaptation. However, the dN/dS rate ratio cannot be used to detect simultaneous increases or simultaneous decreases in nonsynonymous and synonymous rates (60). To address this issue and to further dissect and explore the molecular adaptation process, the rate of substitution in every branch in a tree can be divided into expected nonsynonymous (E[N]) and expected synonymous (E[S]) substitution rates. These absolute rate estimates reflect the respective contributions of E[N] and E[S] substitution rates to the overall substitution rate for a particular branch (17). These rates are uncorrected for the number of possible nonsynonymous and synonymous alterations (i.e., the number of possible nonsynonymous alterations is higher than the number of possible synonymous alterations). Consequently, direct comparisons between E[N] and E[S] estimates may be difficult to interpret. However, relative differences between, e.g., patient groups in E[N] or E[S] estimates can still be explored. Analyses of E[N] and E[S] and divergence plots were performed as described by Lemey et al. (17). Briefly, 200 random trees from the HPM analysis (the model settings included HPM performed with a relaxed clock, constant population and constant size, and the GY94 codon model [58]) were selected for each individual to determine E[N] and E[S] rates in HyPhy 2.2.0 (61). The accumulated divergence was estimated and plotted over time using the weighted average rate. The analysis was performed using a relaxed clock model because a strict clock model, by definition, would assume a linear relationship.

### Analysis of selected sites.

A Renaissance counting approach, as implemented in BEAST v1.8.1(55), was employed to estimate the ratio of nonsynonymous and synonymous substitutions at each codon site, allowing identification of sites that were under positive or negative selection (62). A dN/dS value of 1 suggests neutral selection, a dN/dS value of >1 suggests positive selection, and a dN/dS value of <1 suggests negative selection. The analysis was performed using a strict clock model with a constant population size model as the tree prior and a triply partitioned (first, second, and third) HKY nucleotide substitution model (56). Significant positive or negative selection was considered to have occurred when the HPD interval did not include a value of 1.

### HIV-2/SIVsm conservation analysis.

The *env* reference sequences of HIV-2 and SIVsm (*n* = 61 and *n* = 54, respectively) were downloaded from the Los Alamos Database and aligned using the Clustal algorithm. A strict consensus sequence was obtained using Consensus Maker (available at https://www.hiv.lanl.gov/). Amino acid positions with 100% amino acid conservation were considered to represent HIV-2/SIVsm conserved positions (see Table S8 at https://dochub.com/patrik-medstrand/zqp8z8/supplemntaryinformation_palm_mbio_ver2_180822?dt=Nx2HKD__sa_SKWC-XzVE).

### Surface accessibility.

The solvent accessibility surface area of the HIV-2 gp125 core (PDB accession number 5CAY) was quantified using the Web server running DSSP (http://www.cmbi.ru.nl/xssp/) (63). The relative solvent accessibility (RSA) was calculated as the accessible area divided by the maximum accessible area of the amino acid in extended tripeptide confirmation (Gly-X-Gly), as previously described (64). Residues with an RSA value of ≥5% were considered to be surface exposed (65). The HIV-2 Env protein (PDB accession number 5CAY) was visualized and surface exposed, and positively selected amino acids were edited using UCSF Chimera (https://www.cgl.ucsf.edu/chimera/).

### Statistical analysis.

Statistical analyses were performed in IBM SPSS Statistics 21 using the two-tailed Mann-Whitney U test (M-W) for continuous variables, the two-tailed Fisher’s exact test (FET) for categorical variables, Spearman’s rho for correlation analyses, and Friedman’s test for comparisons of multiple groups. Results from the Friedman’s test showing statistical significance were then analyzed by the Bonferroni-corrected Wilcoxon signed rank test for pairwise comparisons of groups.

### Ethics approval and consent to participate.

The study was approved by the ethics committees of the government of Guinea-Bissau; the University of Lund, Sweden; and the Karolinska Institute, Stockholm. Study participants were counselled and provided informed oral consent.

Supplementary information (including supplementary text, figures, and tables) has been deposited at https://dochub.com/patrik-medstrand/zqp8z8/supplemntaryinformation_palm_mbio_ver2_180822?dt=Nx2HKD__sa_SKWC-XzVE.

### Data availability.

The data sets generated and analyzed during the current study are available in the GenBank repository (accession numbers: KM390990 to KM391398).
